# A comparison of self-report and antiretroviral detection to inform estimates of antiretroviral therapy coverage, viral load suppression and HIV incidence in Kwazulu-Natal, South Africa

**DOI:** 10.1186/s12879-017-2740-y

**Published:** 2017-09-29

**Authors:** Helena Huerga, Fisseha Shiferie, Eduard Grebe, Ruggero Giuliani, Jihane Ben Farhat, Gilles Van-Cutsem, Karen Cohen

**Affiliations:** 1 Clinical Research, Epicentre, Paris, France; 2 South African DST-NRF Centre of Excellence in Epidemiological Modelling and Analysis (SACEMA), Stellenbosch University, Stellenbosch, South Africa; 3 Medical Department, Médecins Sans Frontières, Cape Town, South Africa; 4 Centre for Infectious Disease Epidemiology and Research, University of Cape Town, Cape Town, South Africa; 5 Division of Clinical Pharmacology, University of Cape Town, Cape Town, South Africa

**Keywords:** HIV, ART, Self-report, ARV detection, Antiretroviral coverage, HIV incidence, Viral suppression

## Abstract

**Background:**

Accurately identifying individuals who are on antiretroviral therapy (ART) is important to determine ART coverage and proportion on ART who are virally suppressed. ART is also included in recent infection testing algorithms used to estimate incidence. We compared estimates of ART coverage, viral load suppression rates and HIV incidence using ART self-report and detection of antiretroviral (ARV) drugs and we identified factors associated with discordance between the methods.

**Methods:**

Cross-sectional population-based survey in KwaZulu-Natal, South Africa. Individuals 15–59 years were eligible. Interviews included questions about ARV use. Rapid HIV testing was performed at the participants’ home. Blood specimens were collected for ARV detection, LAg-Avidity HIV incidence testing and viral load quantification in HIV-positive individuals. Multivariate logistic regression models were used to identify socio-demographic covariates associated with discordance between self-reported ART and ARV detection.

**Results:**

Of the 5649 individuals surveyed, 1423 were HIV-positive. Median age was 34 years and 76.3% were women. ART coverage was estimated at 51.4% (95%CI:48.5–54.3), 53.1% (95%CI:50.2–55.9) and 56.1% (95%CI:53.5–58.8) using self-reported ART, ARV detection and both methods combined (classified as ART exposed if ARV detected and/or ART reported) respectively. ART coverage estimates using the 3 methods were fairly similar within sex and age categories except in individuals aged 15–19 years: 33.3% (95%CI:23.3–45.2), 33.8% (95%CI:23.9–45.4%) and 44.3% (95%CI:39.3–46.7) using self-reported ART, ARV detection and both methods combined. Viral suppression below 1000cp/mL in individuals on ART was estimated at 89.8% (95%CI:87.3–91.9), 93.1% (95%CI:91.0–94.8) and 88.7% (95%CI:86.2–90.7) using self-reported ART, ARV detection and both methods combined respectively. HIV incidence was estimated at 1.4 (95%CI:0.8–2.0) new cases/100 person-years when employing no measure of ARV use, 1.1/100PY (95%CI:0.6–1.7) using self-reported ART, and 1.2/100PY (95%CI:0.7–1.7) using ARV detection. In multivariate analyses, individuals aged 15–19 years had a higher risk of discordance on measures of ARV exposure (aOR:9.4; 95%CI:3.9–22.8), while migrants had a lower risk (aOR:0.3; 95%CI:0.1–0.6).

**Conclusions:**

In KwaZulu-Natal, the method of identifying ARV use had little impact on estimates of ART coverage, viral suppression rate and HIV incidence. However, discordant results were more common in younger individuals. This may skew estimates of ART coverage and viral suppression, particularly in adolescent surveys.

**Electronic supplementary material:**

The online version of this article (10.1186/s12879-017-2740-y) contains supplementary material, which is available to authorized users.

## Background

Accurately identifying individuals who are on antiretroviral therapy (ART) in HIV surveys is important to determine ART coverage and the proportion of those on ART who are virally suppressed. In addition, antiretroviral (ARV) use is often included in recent infection testing algorithms (RITAs) used to estimate incidence, to ensure that patients on ART are not misclassified as recently infected.

ART exposure can be identified by including questions on prior and current ART use in survey questionnaires (self-report), or by detection of antiretroviral drugs (ARVs) in blood. Self-report has commonly been used in surveys to identify individuals on ART [1–5] but accuracy cannot be verified without an objective measure. ARV detection in dried blood spots (DBS) is an objective method to identify patients with recent ART exposure, but may not identify individuals on ART who have poor or irregular adherence to ART.

We used data from a population-based HIV survey conducted in Mbongolwane and Eshowe, KwaZulu-Natal (KZN), South Africa [6]. In this analysis we compared estimates of ART coverage and viral load suppression rates when we used self-report and/or ARV detection to identify individuals on ART. We assessed the agreement between the two methods of identifying individuals on ART and the socio-demographic covariates associated with discordance between ART self-report and ARV detection. Finally, we determined the impact of including ART use, as ascertained by these two methods, on HIV incidence estimation.

## Methods

### Design and population

This study is part of a cross-sectional population-based survey conducted from July to October 2013 that aimed to assess HIV prevalence, incidence and the HIV cascade of care in Mbongolwane and Eshowe, KZN, [6]. Individuals aged 15–59 years old living in the area were eligible for inclusion in the survey.

### Procedures

Participants were interviewed at home using a structured questionnaire. Interviews were carried out in an isolated part of the dwelling to preserve confidentiality. We collected socio-demographic information and history of HIV clinical care. The questionnaire included three questions about whether or not the participant was on ART: ‘Have you ever initiated ART, antiretroviral drugs against HIV/AIDS?’, ‘When did you first start antiretroviral drugs?’, ‘Are you still receiving antiretroviral drugs?’ HIV testing was performed at the participants’ home using the Determine Rapid HIV-1/2 Antibody test kit, as a screening test, followed by the Unigold Rapid HIV test kit. HIV-positivity was confirmed by ELISA. Venous blood specimens were collected for single-well limiting antigen avidity enzyme immunoassay (LAg-Avidity EIA) and Nucleic Acid Amplification Testing (NAAT). DBS were prepared at the laboratory to test for the presence of ARVs. We performed qualitative testing for the presence of nevirapine, efavirenz and lopinavir by liquid chromatography tandem mass spectrometry with a limit of quantification of 0.04 micrograms/ml for all drugs. The median time window from last drug intake to a negative detection depends on the drug and ranges from around 24 h for lopinavir up to 7 days for efavirenz and nevirapine [7–9]. Viral load was quantified using a NucliSens EasyQ HIV-1 v2.0 assay from Biomerieux. NAAT testing was performed on 5 member pools using Roche AMPLISCREEN, with positive pools reflexed to individual specimen testing using the Roche CAP/CTM method. HIV-1 genotyping was carried out at the Centre for AIDS Prevention and Research in South Africa (CAPRISA) on samples from participants on ART with viral load higher than 1000 copies/ml, using the TRUGENE HIV-1 Genotyping assay from Siemens Healthcare Diagnostics. Genotypic resistance was interpreted according to the Stanford University algorithm for HIV drug resistance.

### Definitions

Self-reported ART: Individuals who reported that they had “ever initiated ART” and were “still receiving ART”.

ARV detected: Nevirapine, efavirenz or lopinavir detected in DBS.

Combined method to identify ART exposure: Categorized as on ART if either ART was self-reported or ARV was detected.

ART discordance: Individuals identified as on ART by one measure but not the other. There were 2 categories of discordance: 1. Individuals who reported being on ART but had no detectable ARV (these likely represent individuals on ART with irregular adherence who did not take any ART in the 24 h before, with a longer window for efavirenz). 2. Individuals in whom an ARV was detected who did not report taking ART (these are likely individuals who chose not to disclose being on ART).

ART coverage among all HIV-positive individuals: proportion of HIV-positive individuals on ART among all HIV-positive individuals.

ART coverage among individuals qualifying for ART: proportion of HIV-positive individuals on ART among individuals whether already on ART or qualifying for ART. Criteria for initiation of ART by National Guidelines at the time of the survey were: CD4 < 350cells/μl, pregnant and breastfeeding women.

Viral load suppression rate: viral load below 1000 cp/mL among those identified as being on ART.

Mobility categories: Resident: individuals belonging to the household who had not moved their residency in the previous 10 years. Migrant: individuals belonging to the household who had moved their residency in the previous 10 years. Visitor: individuals not belonging to the household who slept in the household the night before the day of the interview.

### Data analyses

Descriptive analyses are presented with 95% confidence intervals (95%CI). Three estimates of ART coverage and viral suppression were calculated using self-reported ART, ARV detection and the combined method to identify ART exposure. HIV incidence was calculated by the method of Kassanjee et al. [10]. The case definition for ‘recent infection’ was: either acute infection (antibody negative but NAAT positive) or recent on the compound rule of LAg-Avidity EIA normalized optical density below 1.5, viral load above 100 cp/mL and not on ART. Three estimates of HIV incidence were calculated: employing no measure of ART use and employing either self-reported ART or ARV detection to identify the individuals not on ART. We estimated Mean Duration of Recent Infection (MDRI) for this RITA at 184 days (95% CI: 159–219), based on a subtype C-specific estimate for the LAg-Avidity EIA and the sensitivity of the screening algorithm [11]. The False Recent Rate (FRR) was estimated in a similar context at 0.2% [12].

Interrater agreement (kappa, ƙ) was used to quantify agreement between self-report and ARV detection. Multivariate logistic regressions were used to explore associations between participant characteristics and discordance between self-reported ART and ARV detection with 3 outcomes: (1) ARV detected and not self-reported, (2) ART self-reported and not detected, (3) any ART discordance (ARV detected and not reported or ART reported and not detected). Factors included in the model were: sex (women, men), age (15–19, 20–34, 35–44, 45–59 years), marital status (never married, married/living together, divorced/separated/widowed), level of education (primary or less, secondary or more), area of residence (urban, rural), mobility (resident, migrant, visitor) and employment (employed, not employed). After selecting factors associated with discordance with *p* < 0.25 in the univariate analyses, we applied a step-wise decreasing strategy to select the variables in the multivariate analyses. Sex, age and variables with *p*-value < 0.10 were kept in the final model. Data were analyzed using Stata 13 (Stata Corp., College Station, Texas, USA). Incidence analyses were performed using the *inctools* R package [13].

## Results

### Description of the survey population

The survey included 5649 individuals of which 1423 were found to be HIV-positive. Median age of HIV positive participants was 34 years (IQR: 27–42) and 1085 (76.3%) were women, 345 (24.3%) lived in couple, 686 (48.2%) had completed at least secondary education, 1142 (80.3%) lived in rural areas, 225 (15.8%) were migrants, 711 (50.0%) were unemployed (Table 1).

**Table 1:** Socio-demographic characteristics of the HIV-positive participants

	Women	Men	Total
	(*N* = 1085)	(*N* = 338)	(*N* = 1423)
	n (%)	n (%)	n (%)
Age, years			
15–19	58 (5.4)	12 (3.6)	70 (4.9)
20–34	537 (49.5)	140 (41.4)	677 (52.5)
35–44	281 (25.9)	105 (31.1)	386 (27.1)
45–59	209 (19.3)	81 (24.0)	290 (20.4)
Marital Status			
Never Married	753 (69.4)	236 (70.0)	989 (69.6)
Married/Living Together	265 (24.4)	80 (23.7)	345 (24.3)
Divorced/Separated/Widowed	67 (6.2)	21 (6.2)	88 (6.2)
Education			
No schooling	96 (8.9)	31 (9.2)	127 (8.9)
Primary	448 (41.3)	161 (47.8)	609 (42.8)
Secondary	513 (47.3)	137 (40.7)	650 (45.7)
Tertiary	28 (2.6)	8 (2.4)	36 (2.5)
Place residence			
Urban	202 (18.6)	79 (76.6)	281 (19.8)
Rural	883 (81.4)	79 (23.4)	1142 (80.3)
Mobility			
Residents	876 (80.7)	265 (78.4)	1141 (80.2)
Migrants	170 (15.7)	55 (16.3)	225 (15.8)
Visitors	39 (3.6)	18 (5.3)	57 (4.0)
Occupation			
Employed	520 (47.9)	192 (56.8)	712 (50.0)
Not employed	565 (52.1)	146 (43.2)	711 (50.0)

### ART coverage estimates

ART coverage among all HIV-positive individuals was estimated at 51.4% (95%CI: 48.5–54.3) of HIV-positive individuals using self-reported ART, 53.1% (95%CI: 50.2–55.9) using ARV detection and 56.1% (95%CI: 53.5–58.8) using with both methods combined (Table 2). ART use was reported by 712/1385 of HIV-positive individuals who completed the structured questionnaire. Of 1396 with specimens tested, 741 had ARVs detected in DBS. Combining the two methods we calculated that 798/1423 participants were on ART.

**Table 2:** ART coverage estimates by sex and age using self-report, ARV detection in DBS and both methods combined among all HIV-positive individuals and among individuals qualifying for ART by National Guidelines at the time of the survey

	Self-report	ARV detection	Combined measure^a^
	% (95%CI)	% (95%CI)	% (95%CI)
All HIV-positive	51.4 (48.5–54.3)	53.1 (50.2–55.9)	56.1 (53.5–58.8)
Women	52.6 (49.5–55.6)	55.4 (52.5–58.2)	57.8 (54.9–60.7)
Men	47.7 (41.2–54.3)	45.8 (39.9–51.8)	50.6 (44.4–56.8)
15–19 years	33.3 (23.3–45.2)	33.8 (23.9–45.4)	44.3 (33.0–56.2)
20–34 years	37.2 (33.6–41.0)	41.1 (37.3–44.9)	43.0 (39.3–46.7)
35–44 years	67.8 (62.6–72.6)	67.6 (62.7–72.2)	72.5 (67.8–76.8)
45–59 years	66.6 (61.5–71.2)	66.1 (60.6–71.2)	67.6 (62.4–72.4)
Qualifying for ART^b^	74.8 (71.7–77.6)	75.0 (72.0–77.8)	78.1 (75.2–80.7)
Women	77.8 (75.0–80.5)	78.5 (75.8–81.0)	81.1 (78.5–83.5)
Men	65.7 (58.1–72.5)	63.9 (56.6–70.5)	68.7 (61.4–75.2)
15–19 years	63.9 (47.6–77.5)	59.0 (42.1–74.0)	73.8 (58.6–84.9)
20–34 years	62.3 (57.5–66.9)	64.8 (60.1–69.2)	67.4 (62.8–71.6)
35–44 years	85.3 (80.1–89.3)	84.8 (80.0–88.7)	88.6 (84.0–92.0)
45–59 years	84.1 (79.2–88.1)	83.6 (78.0–88.1)	84.5 (79.2–88.6)

^a^Combined measure: categorized as ART exposed by at least one method

^b^Individuals qualifying for ART by National Guidelines at the time of the survey: initiated on ART, not on ART with CD4 < 350cells/μl, pregnant and breastfeeding women

ART coverage among people qualifying for ART at the time of the survey was estimated at 74.8% (95%CI: 71.7–77.6) using self-reported ART, 75.0% (95%CI: 72.0–77.8) using ARV detection and 78.1% (95%CI: 75.2–80.7) using with both methods combined. ART coverage estimates using the 3 methods were generally fairly similar within sex and age categories except in individuals aged 15–19 years. In this age group, coverage was higher when both methods were combined, compared to the estimates by ARV detection or by self-report alone, although this difference was not statistically significant. Using any of the 3 methods, ART coverage was higher in individuals aged 35–59 years compared to those aged 15–34 years (*p* < 0.001 for all 3 methods).

Of those with ARV detected, 581 (78.4%) were on efavirenz-containing ART, 105 (14.2%) on nevirapine-containing ART, and 61 (8.2%) on lopinavir-containing ART. In 6 patients more than one drug was detected: 3 with nevirapine and lopinavir, 2 with nevirapine and efavirenz, 1 with efavirenz and lopinavir.

### Viral suppression estimates and HIV-1 ARV resistance mutations

Viral suppression rates in individuals on ART was 89.8% (95%CI: 87.3–91.9) using self-report, 93.1% (95%CI: 91.0–94.8) using ARV detection and 88.7% (95%CI: 86.2–90.7) with both methods combined, respectively (Table 3). Viral suppression was lower in individuals who reported being on ART but who had no ARV detected, 25.0% (13/52) compared to participants who did not report ART but had ARV detected, 70.2% (40/57). Among the 30 individuals aged 15–19 years with ART reported or ARVs detected, 8 reported being on ART but ARV were not detected and in 7 individuals ARVs were detected but not reported. Thus, in individuals aged 15–19 years, viral suppression was lower among those who reported being on ART than among those in whom ARV was detected: 56.5% (95%CI: 35.6–75.4) vs 87.0% (95%CI: 63.9–95.2).

**Table 3:** Viral suppression below 1000 cp/mL by sex and age using self-report, ARV detection and both methods combined among HIV-positive individuals on ART

	Self-report	ARV detection	Combined measure^a^
	% (95%CI)	% (95%CI)	% (95%CI)
Women	90.4 (87.7–92.6)	93.4 (91.1–95.1)	89.6 (87.1–91.6)
Men	87.7 (81.5–92.1)	92.1 (86.0–95.7)	85.2 (78.9–89.9)
15–19 years	56.5 (35.6–75.4)	87.0 (63.9–95.2)	67.7 (49.5–81.8)
20–34 years	88.0 (83.4–91.5)	91.5 (86.9–94.6)	86.9 (82.2–90.4)
35–44 years	89.8 (85.1–93.1)	92.6 (88.6–95.3)	87.8 (83.3–91.2)
45–59 years	96.6 (92.5–98.2)	96.8 (93.2–98.6)	95.9 (92.0–97.9)
All individuals	89.8 (87.3–91.9)	93.1 (91.0–94.8)	88.7 (86.2–90.7)

^a^Combined measure: at least one method determining positive ART exposure

Resistances tests were performed in the 54 patients who reported being on ART for more than 6 months and had viral load greater than 1000 copies/mL. Among the participants who reported ART, 61.1% (33/54) had at least one resistance mutation while this proportion increased to 72.4% (21/29) in individuals who had ARVs detected. There were NNRTI resistance mutations in 31/54 (57.4%), NRTI mutations in 25/54 (46.3%) and PI mutations in 5/62 (9.3%) (Additional file 1: Table S1). Among the participants with reported ART and no ARVs detected, 48.0% (12/25) had at least one resistance mutation: 7 to NRTIs, 10 to NNRTIs and 4 to PIs. Among participants taking NNRTI’s as per blood test, 74.1% (20/27) had a positive resistance test to any of the drugs in the regimen. The 4 participants taking LPV as per DBS test did not have any resistance mutation detected to PI.

### HIV incidence estimates

HIV incidence was estimated at 1.4 new cases per 100PY (95%CI: 0.8–2.0) when employing no measure of ARV use, 1.1/100PY (95%CI: 0.6–1.7) using reported ART, and 1.2/100PY (95%CI: 0.7–1.7) using ARV detection (Table 4). Incidence estimates were similar using the 2 methods, with differences in point estimates constituting a small fraction of the span of confidence intervals. This analysis did not provide strong evidence for preferring one measure over the other.

**Table 4:** HIV incidence estimates by sex and age without an ARV measure, using ART self-report and using ARV detection

	No ARV measure	ART reported	ARV detected
	/100PY (95%CI)	/100PY (95%CI)	/100PY (95%CI)
Women 15–29	2.4 (1.2–3.7)	2.2 (1.1–3.5)	2.4 (1.2–3.6)
Men 15–29	0.9 (0.2–1.7)	0.7 (0.1–1.4)	0.7 (0.1–1.4)
Women 30–59	0.8 (0.0–1.7)	0.1 (0.0–0.8)	0.1 (0.0–0.8)
Men 30–59	0.6 (0.0–1.9)	0.6 (0.0–1.9)	0.6 (0.0–1.9)
All individuals	1.4 (0.8–2.0)	1.1 (0.6–1.7)	1.2 (0.7–1.7)

We estimated the maximum improvement in the precision of the incidence estimate that the inclusion of any ARV use measure in the RITA may achieve. Assuming an FRR of 0.5% for the RITA without an ARV measure (substantially higher than our best estimate of 0.2%), the complete elimination of residual false recency, and no impact on MDRI, the relative standard error of the overall incidence estimate would decline from 20.8% to 17.9%. The potential benefit is therefore modest at best.

### Discordance between self-report and ARV detection

Of the 1358 individuals with both self-report and ARV detection information, 655 (48.2%) had ARVs detected and reported being on ART, 58 (4.3%) had ARVs detected but did not report being on ART (non-disclosure) and 52 (3.8%) reported being on ART but did not have ARVs detected (non-adherence) (Additional file 2: Table S2).

There was non-disclosure of ART use in 8.1% (95%CI: 6.3–10.4) of the participants. Non-disclosure was higher at younger ages: 31.8% (95%CI: 15.5–54.3), 11.5% (95%CI: 7.9–16.3), 7.2% (95%CI: 4.6–11.1), 2.1% (95%CI: 0.8–5.4), in individuals aged 15–19, 20–34, 35–44 and 45–59 years respectively (Chi^2^ test for trend *p* < 0.001).

Conversely, 7.4% (95%CI: 5.7–9.5) of the participants reporting ART did not have ARVs detected, suggesting poor recent adherence to ART. The proportion with discordance was higher in individuals 15–19 years compared to individuals in the other group ages: 34.8% (95%CI: 18.0–56.5) vs 7.4% (95%CI: 4.7–11.6), 8.3% (95%CI: 5.2–12.9) and 2.7% (95%CI: 1.1–6.1) in individuals aged 20–34, 35–44 and 45–59 years respectively (Chi^2^ test for trend *p* < 0.001).

Among individuals who reported being on ART, median time since ART initiation was longer for individuals who had ARVs detected compared to those for whom ARVs were not detected: 33.8 vs 13.9 months.

Agreement between ART self-reported and ARV detection was 91.9% (kappa = 0.84, 95%CI: 0.81–0.87). There was no difference in the agreement between self-report and ARV detection among male and female participants, 92.0% (kappa = 0.84, 95%CI: 0.78–0.90) and 91.9% (kappa = 0.84, 95%CI: 0.80–0.87) respectively. Agreement between the 2 methods of determining ART exposure was lower in individuals aged 15–19 years: 77.6% (kappa = 0.50, 95%CI: 0.28–0.72) vs 92.6% (kappa = 0.85, 95%CI: 0.80–0.89), 89.5% (kappa = 0.76, 95%CI: 0.69–0.83) and 96.8% (kappa = 0.93, 95%CI: 0.88–0.97) in individuals aged 20–34, 35–44 and 45–59 years respectively.

In multivariate logistic regressions, younger individuals were at higher risk of discordant results (Table 5). Individuals younger than 45 years and particularly those aged 15–19 years had the highest risk of all types of discordance (Table 5) while migrants were less likely to have discordant results. Individuals with an education of lower than secondary school who had reported ART were more likely to test negative for ARVs than those with higher levels of education.

**Table 5:** Factors associated ART discordance using self-report and ARV detection in HIV-positive individuals

	ART detected & not reported						ART reported & not detected						ART any discordance					
	(*N* = 1358)						(*N* = 1358)						(*N* = 1358)					
	OR	95%CI	*p*	a OR	95%CI	*p*	OR	95%CI	*p*	a OR	95%CI	*p*	OR	95%CI	*p*	aOR	95%CI	*p*
Sex																		
Women	1			1			1			1			1			1		
Men	0.6	0.3–1.2	0.15	0.6	0.3–1.3	0.19	1.6	0.9–2.8	0.15	1.6	0.9–2.9	0.14	1.0	0.6–1.6	0.97	1.0	0.7–1.7	0.83
Age group (years)																		
45–59	1			1			1			1			1			1		
35–44	3.6	1.2–10.7	0.01	3.6	1.2–10.8	0.02	3.3	1.2–9.0	0.02	3.9	1.4–10.4	<0.01	3.6	1.7–7.5	<0.01	3.6	1.7–7.7	<0.01
20–34	3.3	1.2–9.5	<0.01	3.3	1.2–9.7	0.02	1.6	0.6–4.4	0.35	2.4	0.9–6.8	0.01	2.4	1.2–5.0	0.02	2.6	1.2–5.4	0.01
15–19	8.1	2.3–28.7	<0.01	8.1	2.3–28.6	<0.01	7.5	2.4–23.9	<0.01	11.5	3.5–38.1	<0.01	8.8	3.7–21.1	<0.01	9.4	3.9–22.8	<0.01
Marital status																		
Never Married	1						1						1					
Married/Living Together	1.2	0.7–2.1	0.58				0.8	0.4–1.5	0.47				1.0	0.6–1.5	0.91			
Divorced/Separated/Widowed	1.2	0.4–3.5	0.70	–	–	–	0.6	0.1–2.5	0.46	–	–	–	0.9	0.4–2.1	0.80	–	–	–
Education																		
2ary or more	1						1						1					
1ary or less	0.6	0.4–1.1	0.11	–	–	–	1.8	0.0–3.2	0.05	2.0	1.1–3.7	0.03	1.0	0.7–1.5	0.85	–	–	–
Living area																		
Rural	1						1						1					
Urban	0.4	0.2–1.1	0.07	–	–	–	0.9	0.5–1.9	0.82	–	–	–	0.7	0.4–1.1	0.14	–	–	–
Mobility																		
Resident	1			1			1			1			1			1		
Visitor	0.8	0.2–3.4	0.78	0.8	0.2–3.4	0.77	0.9	0.2–3.8	0.87	0.8	0.2–3.3	0.72	0.8	0.3–2.3	0.75	0.8	0.3–2.2	0.64
Migrant	0.4	0.1–1.1	0.06	0.4	0.1–1.0	0.06	0.2	0.0–0.8	0.03	0.2	0.0–0.9	0.03	0.3	0.1–0.7	<0.01	0.3	0.1–0.6	<0.01
Employment																		
Employed	1						1						1					
Unemployed	1.0	0.6–1.7	0.96	–	–	–	0.9	0.7–2.0	0.62	–	–	–	0.9	0.6–1.4	0.76	–	–	–

## Discussion

We found that estimates of antiretroviral coverage were generally similar when using self-report and ARV detection to identify individuals on ART. Overall, there was good concordance between self-report of ART use and detection of ARVs in this study population. However, discordance was higher in individuals aged 15–19 years suggesting higher proportions of poor recent adherence and non-disclosure in young people. As a result, estimates of ART coverage in this group were sensitive to the measure of ART exposure used. Individuals who had moved residence in the last ten years were less likely to have discordance than permanent residents. The reason for this is unclear and qualitative research is needed to explore differences regarding barriers to both disclosure and adherence in these populations.

Rates of viral suppression were slightly higher using ARV detection to identify those on ART. This finding is expected since those with ARVs detected are likely to be currently adherent to therapy. On the other hand, individuals reporting ART with no detectable ARV are likely to be poorly adherent and viral suppression was very low in this group. Median time since ART initiation was longer for individuals who had ARVs detected which may be due to patients in this cohort struggling with adherence particularly in the period immediately after ART commencement, or because poorly adherent patients default therapy. Estimates of suppression in adolescents ranged widely depending on the method of identifying ART exposure. The prevalence of resistance among individuals on ART was also sensitive to the method used to determine the denominator (higher for ARV detection).

We found that the method of identifying ARV use had little impact on incidence estimates. Including ARV use in the RITA had only a modest impact on precision of incidence estimates.

Measures of ARV use are frequently included in recent infection testing algorithms (RITAs) on the intuition that this would reduce the false recent rate (FRR) and improve the precision of the incidence estimates. Context-specific FRR estimates for an algorithm consisting of LAg-Avidity EIA and viral load in a setting similar to the study population have been estimated as low as 0.2% [12]. Furthermore, benchmarking data are not available for quantifying the impact of including a measure of ARV use on the mean duration of recent infection (MDRI) and FRR. It is therefore questionable whether such measures should be included in RITAs that already contain a viral load measurement. However, with early treatment becoming increasingly common and with higher treatment coverage, larger numbers of individuals on treatment but with detectable viral loads, continuing to study the impact of ARV ascertainment on RITA properties will be essential.

Based on these data, either method of ART exposure ascertainment could be used for estimation of ART coverage and viral suppression for the population as a whole. However, we found that discordant results were more common in younger individuals (9 folds higher risk). This may skew estimates of ART coverage and viral suppression, particularly in adolescent surveys. Based on our data, both non-disclosure and poor recent adherence are common in this group.

Use of a combination of methods may be useful for particular study questions: for example this would allow for exploration of adherence patterns within a survey, and for exploration of the relationship between adherence, viral suppression and viral resistance. Including ARV detection also allows for characterization of patterns of ART regimen use within the surveyed population.

This study has limitations. Adolescents formed a small proportion of individuals in the sample, and we therefore lacked power for precise estimates in this group. We did not have access to hospital and dispensing records, which would provide an objective confirmation of long-term ART.

## Conclusions

In conclusion, in KZN the method of identifying ARV use had little impact on estimates of ART coverage, viral suppression rate and HIV incidence. The method for determining ART exposure should be selected based on the primary study questions. Surveys focused on young individuals, particularly adolescents, should consider using the combination of self-report and ARV detection, so that estimates are not skewed by non-disclosure or poor adherence.

## Additional files

Additional file 1: Table S1. Resistance profile in individuals who reported being on ART for more than 6 months and with viral load ≥1000cp/mL. (DOCX 14 kb)

Additional file 2: Table S2. ART self-report and ARV detection by sex and age. (DOCX 15 kb)

## Abbreviations

ART: Antiretroviral therapy (ART)
ARV: Antiretroviral (ARV)
ARVs: Antiretroviral drugs (ARVs)
DBS: Dried blood spots (DBS)
KZN: KwaZulu-Natal (KZN)
LAg-Avidity EIA: Limiting antigen avidity enzyme immunoassay
NAAT: Nucleic Acid Amplification Testing (NAAT)
RITAs: Recent infection testing algorithms

## References

[1] Kranzer K, Lawn SD, Johnson LF, Bekker L-G, Wood R. Community viral load and CD4 count distribution among people living with HIV in a south African township: implications for treatment as prevention. J Acquir Immune Defic Syndr. 2013 [cited 2016 Apr 29];63:498–505. Available from: http://www.pubmedcentral.nih.gov/articlerender.fcgi?artid=4233323&tool=pmcentrez&rendertype=abstract. 10.1097/QAI.0b013e318293ae48 PMC4233323 23572010

[2] Cherutich P, Kim AA, Kellogg TA, Sherr K, Waruru A, De Cock KM, et al. Detectable HIV viral load in Kenya: data from a population-based survey. PLoS One. 2016 [cited 2016 Jun 30];11:e0154318. Available from: http://www.ncbi.nlm.nih.gov/pubmed/27192052. 10.1371/journal.pone.0154318 PMC4871583 27192052

[3] Maman D, Chilima B, Masiku C, Ayouba A, Masson S, Szumilin E, et al. Closer to 90-90-90. The cascade of care after 10 years of ART scale-up in rural Malawi: a population study. J Int AIDS Soc. [Internet]. 2016 [cited 2016 Jun 30];19:20673. Available from: http://www.ncbi.nlm.nih.gov/pubmed/26894388. 10.7448/IAS.19.1.20673 PMC4760102 26894388

[4] Odhiambo JO, Kellogg TA, Kim AA, Ng’ang’a L, Mukui I, Umuro M, et al. Antiretroviral treatment scale-up among persons living with HIV in Kenya: results from a nationally representative survey. J Acquir Immune Defic Syndr. [Internet]. 2014 [cited 2015 Sep 10];66 Suppl 1:S116-S122. Available from: http://www.ncbi.nlm.nih.gov/pubmed/24732815. 10.1097/QAI.0000000000000122 PMC4786176 24732815

[5] Bicego GT, Nkambule R, Peterson I, Reed J, Donnell D, Ginindza H, et al. Recent patterns in population-based HIV prevalence in Swaziland. PLoS One [Internet]. 2013 [cited 2016 Apr 29];8:e77101. Available from: http://www.pubmedcentral.nih.gov/articlerender.fcgi?artid=3797108&tool=pmcentrez&rendertype=abstract. 10.1371/journal.pone.0077101 PMC3797108 24143205

[6] Huerga H, Van Cutsem G, Ben Farhat J, Reid M, Bouhenia M, Maman D, et al. Who needs to be targeted for HIV testing and treatment in KwaZulu-Natal? Results from a population-based survey. J Acquir Immune Defic Syndr. [Internet]. 2016 [cited 2016 Jun 30]; Available from: http://www.ncbi.nlm.nih.gov/pubmed/27243903. 10.1097/QAI.0000000000001081 PMC5172512 27243903

[7] Boffito M, Else L, Back D, Taylor J, Khoo S, Sousa M, et al. Pharmacokinetics of atazanavir/ritonavir once daily and lopinavir/ritonavir twice and once daily over 72 h following drug cessation. Antivir Ther. [Internet]. 2008 [cited 2016 Sep 2];13:901–907. Available from: http://www.ncbi.nlm.nih.gov/pubmed/19043924. 19043924

[8] Jackson A, Moyle G, Watson V, Tjia J, Ammara A, Back D, et al. Tenofovir, emtricitabine intracellular and plasma, and efavirenz plasma concentration decay following drug intake cessation: implications for HIV treatment and prevention. J Acquir Immune Defic Syndr. [Internet]. 2013 [cited 2016 Sep 2];62:275–281. Available from: http://www.ncbi.nlm.nih.gov/pubmed/23274933. 10.1097/QAI.0b013e3182829bd0 23274933

[9] Mackie NE, Fidler S, Tamm N, Clarke JR, Back D, Weber JN, et al. Clinical implications of stopping nevirapine-based antiretroviral therapy: relative pharmacokinetics and avoidance of drug resistance. HIV Med. [Internet]. 2004 [cited 2016 Sep 2];5:180–184. 10.1111/j.1468-1293.2004.00208.x 15139985

[10] Kassanjee R, McWalter TA, Bärnighausen T, Welte A. A new general biomarker-based incidence estimator. Epidemiology [Internet]. 2012 [cited 2015 Nov 2];23:721–728. Available from: http://www.pubmedcentral.nih.gov/articlerender.fcgi?artid=3500970&tool=pmcentrez&rendertype=abstract. 10.1097/EDE.0b013e3182576c07 PMC3500970 22627902

[11] Grebe, E. HIV Subtypes – MDRI Best Estimates. Presentation to WHO Technical Working Group on HIV Incidence Assays, Boston, MA, 20 February. 2016.

[12] Kassanjee R, Pilcher CD, Busch MP, Murphy G, Facente SN, Keating SM, et al. Viral load criteria and threshold optimization to improve HIV incidence assay characteristics - a CEPHIA analysis. AIDS. 2016;ePub Ahead. 10.1097/QAD.0000000000001209 PMC5024345 27454561

[13] Welte A, Grebe E, McIntosh A, Bäumler P, Ongarello S. inctools: Incidence Estimation Tools. R package, version 1.0.10. http://www.incidence-estimation.org/page/inctools.

