# Supplementary material for: A comparison of self-report and antiretroviral detection to inform estimates of antiretroviral therapy coverage, viral load suppression and HIV incidence in Kwazulu-Natal, South Africa
Source: BMC Infect Dis. 2017 Sep 29;17:653. doi: 10.1186/s12879-017-2740-y (PMC5623964; doi:10.1186/s12879-017-2740-y)
Supplement: Supplementary file 1 — Resistance profile in individuals who reported being on ART for more than 6 months and with viral load ≥1000cp/mL. (DOCX 14 kb) [file 12879_2017_2740_MOESM1_ESM.docx]

Additional file 1: Table S1: Resistance profile in individuals who reported being on ART for more than 6 months and with viral load ≥1000cp/mL

|  | No ARV detected  (N=25)  n (%) | Efavirenz detected  (N=23)  n (%) | Nevirapine detected  (N=4)  n (%) | Lopinavir detected  (N=4)  n (%) | All  (N=54)  n (%) |
| --- | --- | --- | --- | --- | --- |
| Any resistance to NRTI^1^ | 7 (28.0) | 14 (60.9) | 3 (75.0) | 3 (75.0) | 25 (46.3) |
| Any resistance to NNRTI^2^ | 10 (40.0) | 17 (73.9) | 3 (75.0) | 3 (75.0) | 31 (57.4) |
| Any resistance to PI | 4 (16.0) | 1 (4.4) | 0 (0.0) | 0 (0.0) | 5 (9.3) |
| **Any resistance** | **12 (48.0)** | **17 (73.9)** | **3 (60.0)** | **3 (75.0)** | **33 (61.1)** |

1. Individuals with resistances to NRTI: 24 to 3TC, 24 to FTC, 23 to ABC, 10 to DDI, 7 to D4T, 6 to TDF, 2 to AZT.
2. Individuals with resistances to NNRTI: 31 to EFV/NVP, 10 to ETR, 10 to RPV.
