# Supplementary material for: A comparison of self-report and antiretroviral detection to inform estimates of antiretroviral therapy coverage, viral load suppression and HIV incidence in Kwazulu-Natal, South Africa
Source: BMC Infect Dis. 2017 Sep 29;17:653. doi: 10.1186/s12879-017-2740-y (PMC5623964; doi:10.1186/s12879-017-2740-y)
Supplement: Supplementary file 2 — ART self-report and ARV detection by sex and age. (DOCX 15 kb) [file 12879_2017_2740_MOESM2_ESM.docx]

Additional file 2: Table S2: ART self-report and ARV detection by sex and age

|  | ART detection | | |
| --- | --- | --- | --- |
|  | Positive  n (%) | Negative  n (%) | All  n (%) |
| ART self-reported |  |  |  |
| - Women |  |  |  |
| Yes | 517 (50.0) | 35 (3.4) | 552 (53.3) |
| No | 49 (4.7) | 434 (41.9) | 483 (46.7) |
| All | 566 (54.7) | 469 (45.3) | 1035 (100) |
| - Men |  |  |  |
| Yes | 138 (42.7) | 17 (5.3) | 155 (48.0) |
| No | 9 (2.8) | 159 (49.2) | 168 (52.0) |
| All | 147 (45.5) | 176 (54.5) | 323 (100) |
| - 15-19 years |  |  |  |
| Yes | 15 (22.4) | 8 (11.9) | 23 (34.3) |
| No | 7 (10.4) | 37 (55.2) | 44 (65.7) |
| All | 22 (32.8) | 45 (67.2) | 67 (100) |
| - 20-34 years |  |  |  |
| Yes | 224 (35.1) | 18 (2.8) | 242 (37.9) |
| No | 29 (4.5) | 367 (57.5) | 396 (62.1) |
| All | 253 (39.7) | 385 (60.3) | 638 (100) |
| - 35-44 years |  |  |  |
| Yes | 232 (62.7) | 21 (5.7) | 253 (68.4) |
| No | 18 (4.9) | 99 (26.8) | 117 (31.6) |
| All | 250 (67.6) | 120 (32.4) | 370 (100) |
| - 45-59 years |  |  |  |
| Yes | 184 (65.0) | 5 (1.8) | 189 (66.8) |
| No | 4 (1.4) | 90 (31.8) | 94 (33.2) |
| All | 188 (66.4) | 95 (33.6) | 283 (100) |
| - All |  |  |  |
| Yes | 655 (48.2) | 52 (3.8) | 707 (52.1) |
| No | 58 (4.3) | 593 (43.7) | 651 (47.9) |
| All | 713 (52.5) | 645 (47.5) | 1358 (100) |
